# Comparison of clinical outcome of lumbar spinal stenosis surgery in patients with and without osteoporosis: a prospective cohort study

**DOI:** 10.1186/s13018-023-03935-x

**Published:** 2023-06-21

**Authors:** Mashhood Aghajanloo, Ali Abdoli, Jalal Poorolajal, Sajjad Abdolmaleki

**Affiliations:** 1 Department of Neurosurgery, Hamadan University of Medical Sciences, Hamadan, Iran; 2 Department of Epidemiology, School of Public Health, Hamadan University of Medical Sciences, Hamadan, Iran

**Keywords:** Osteoporosis, Lumbar spinal canal stenosis, Clinical outcome, Instrumentation Iran

## Abstract

**Background:**

Osteoporosis is one of the most important risk factors for failure of the spine instrumentation. Management of patients with osteoporosis who requires spinal surgery because of the difficulty in instrument placement and the potential complications is still a challenge. This study was designed to evaluate the clinical outcome of lumbar spinal canal stenosis after instrumentation in patients with and without osteoporosis.

**Methods:**

This prospective cohort study was performed from June 2018 to December 2020, in Be'sat Hospital, Hamadan, Iran. The sample consisted of patients over 50 years old referred to Be'sat Hospital with a diagnosis of lumbar spinal canal stenosis who underwent instrumental surgery (*n* = 107). Based on bone densitometry, the sample was divided into two groups with osteoporosis (*n* = 34) and without osteoporosis (*n* = 73). To collect data, we used a three-part researcher-made questionnaire (demographic information, medical records information, and paraclinical parameters). Statistical analyzes were performed by the Fisher Exact, chi-square, independent t-test, Multiple ANCOVA, Mann–Whitney and the Rank Wilcoxson tests using Stata version 17 software.

**Results:**

The mean age (SD) of patients in the two groups with and without osteoporosis was 67.9 (7.0) and 59.1 (5.1) years, respectively (*p* = 0.001). The results indicated that a significant difference was observed between the two groups in sex (*p* = 0.032), educational status (*p* = 0.001), marital status (*p* = 0.023), employment status (*p* = 0.004), menopausal status (*p* = 0.018), taking corticosteroids (*p* = 0.028), and body mass index (*p* = 0.015). Also, there was a significant difference between two groups in the loosening of instrument (*p* = 0.039), the postoperative pain intensity (*p* = 0.007), fusion (*p* = 0.047), and neurogenic claudication (*p* = 0.003). Based on multiple ANCOVA test, there was not a significant difference between two groups in the clinical and paraclinical charatecristics (*p* > 0.05). The mean (SD) of T-Score in the osteoporosis group was 3.06 (0.37).

**Conclusion:**

This study provides evidence that there is no significant difference in the clinical outcomes of lumbar spine instrumentation due to spinal canal stenosis in patients with and without osteoporosis. Because of the high cost of specific instrumentation developed for patients with osteoporosis and their unavailability, it seems that the use of conventional instrumentation along with complete treatment of osteoporosis can help improve the clinical outcome of surgery in these patients.

## Introduction

Osteoporosis, as the most prevalent bone disorder worldwide, is known for low bone mass, microarchitecture deterioration of bone tissue, and reduced bone strength [1]. Although Osteoporosis is more common in women [2], it is estimated that 50% of women and 20% of men aged > 50 years will experience osteoporosis throughout their lives [1].

Osteoporosis is one of the most important risk factors for fractures and the difficulty in instrument placement due to bone density is reduced [3]. One of the most common areas of low bone mass is the spine [4]. Spinal surgery is seen in 51.3% of women and 14.5% of men aged ≥ 50 years with osteoporosis [4]. Osteoporosis in old age is associated with the risk of compression fractures of the vertebrae [5]. Elderly patients with osteoporosis need more extensive surgeries to fix the spine and are more likely to have surgical complications [6].

Management of patients with osteoporosis who requires spinal surgery because of the difficulty in instrument placement and the potential complications is still a challenge [5]. Patients with osteoporosis are prone to fractures of spine vertebrae and failure of the spine instrumentation, which lead to proximal junctional kyphosis (PJK) and deformity of the spine. In future, the increasing number of elderly patients requiring spinal surgery will make it difficult for spinal surgeons to choose between fixation and fusion [7]. Osteoporosis exposes the patient to fractures, deformity progression, and difficulty in surgical fixation [8].

Given the increase in the elderly population and the spread of sedentary lifestyles among this group, the need for spine surgery and the use of instrumentation for fixation and fusion have increased. One of the common problems of this vulnerable population is reduced bone density and reduced bone strength, including the bones of the spine as the axis of weight-bearing. So, one of the current challenges of spinal surgery is the effect of osteoporosis on the outcomes of spinal instrumentation. Considering that in many medical centers, especially in developing countries like Iran, patients with and without osteoporosis are treated in the same way and so far, no study has been done to compare the outcomes of spinal instrumentation surgery in the two groups. Also, according to our best knowledge, there is limited evidence in this field, the present study was designed to evaluate the clinical outcome of lumbar spinal canal stenosis after instrumentation in patients with and without osteoporosis.

## Method

### Patients

This study was a prospective cohort study that was performed from June 2018 to December 2020 in Be'sat Hospital, Hamadan, Iran. By Purposive sampling method, 107 patients > 50 years old referred to Be'sat Hospital with a diagnosis of lumbar spinal canal stenosis who required spinal surgery were divided into two groups (with osteoporosis, *n* = 34 and without osteoporosis, *n* = 73) based on bone mineral density measurement. The inclusion criteria for the group with osteoporosis were those aged > 50 years old, patients who have osteoporosis based on a bone scan, and spinal canal stenosis up to three levels. The inclusion criteria for the group without osteoporosis were those aged > 50 years old, patients who do not have osteoporosis based on a bone scan, and spinal canal stenosis up to three levels. Patients with a history of spinal surgery and history of any osteoporosis treatment were excluded from the study. All patients expressed their written informed consent to enter the study. This study was approved by the Ethics committee of Hamadan University of Medical Sciences, Iran (IR.UMSHA.REC.1397.175).

### Treatment and follow-up

In this study, the group with osteoporosis underwent osteoporosis treatment for at least one year after spinal surgery. Treatment included a Bisphosphonate drug (Alendronate tablet 70 mg/once weekly), daily Calcium/vitamin D tablet, and nasal calcitonin spray 200 µg/daily) that started three months after surgery. These patients were followed up every six months. At the end of one year, a simple lumbar x-ray was taken.

Although computed tomography (CT) is more sensitive and specific for fusion evaluation, because of cost, radiation side effects, and reluctance to do. So, we considered an x-ray for fusion assessment. Patients with osteoporosis were followed up for some items such as infection, postoperative pain, neurogenic claudication, fusion, loosening, displacement, fracture of the instrument. Patients without osteoporosis were followed up every six months after surgery for the above items.

### Data collection

For collecting data, we used a three-part researcher-made questionnaire (demographic information: age, sex, educational status, marital status, employment status, menopausal status, body mass index (BMI)), medical records information (taking corticosteroids, smoking, alcohol consumption, infection, pre/postoperative pain, neurogenic claudication, fusion, loosening, displacement and fracture of the instrument) and paraclinical parameters (bone densitometry, MRI, CT scan and X-ray).

### Measurements

In the current study, based on the definition of the World Health Organization (WHO), osteoporosis was considered as the presence of T-score < -2.5 SD based on the result of bone scan [9]. In the study, lumbar spinal stenosis was considered as the anteroposterior diameter less than 10 mm of the spinal canal in the axial section [10]. To investigate the fusion was used modified Glassman poster-lateral fusion grading system (1 = Solid bilateral fusion, 2 = Solid unilateral fusion, 3 = Partial bilateral fusion, 4 = Partial unilateral fusion, 5 = No fusion) [11]. Also, neurogenic claudication-induced limitations in walking were assessed by questions that asked patients to estimate the (1) distance (in meter) and (2) time (in minutes or hours) that they could walk without a break on even ground before symptoms become intolerable [12]. Pre/Postoperative pain intensity was assessed using the numeric rating pain scales (RPS). This scale is a segmented numeric version of the visual analog scale (VAS) in which a respondent selects a whole number (0–10 integers) that best reflects the intensity of the pain [13].

We used a simple x-ray (anteroposterior and lateral) to evaluate the fusion, loosening, displacement, and fracture of the instrument. Finally, the rate of infection at the surgical site was determined by sending a culture of the wound discharge to the hospital laboratory in cases that presented with wound discharge.

### Data analysis

The normality of the continuous variables was evaluated using skewness and kurtosis tests for normality. Statistical analyzes were performed by Fisher Exact and chi-square tests for categorical variables and independent t-test and multiple ANCOVA for continuous variables with normal distribution and Mann–Whitney and the Rank Wilcoxson test for continuous variables with abnormal distribution. All analyses were done using Stata software version 17 (College Station, TX, USA). All significance levels were considered less than 0.05.

## Results

The mean age of patients with and without osteoporosis was 67.9 (SD = 7.0) years old and 59.1 (SD = 5.1) years old, respectively (*p* = 0.001). Most patients were women in the two groups. The results indicated that a significant differences were observed between the two groups in sex (*p* = 0.032), educational status (*p* = 0.001), marital status (*p* = 0.023), employment status (*p* = 0.004), menopausal status (*p* = 0.018), taking corticosteroids (*p* = 0.028), and BMI (*p* = 0.015) (Table 1). The T-score in the non-osteoporosis group was ≥ 1, and in the osteoporosis group was ≤ 2.5. The mean (SD) of T-Score in the osteoporosis group was 3.06 (0.37).

**Table 1:** Comparison of characteristics of the study population using the Fisher exact test, the independent t-test and the chi-square test

Categorical variables	Osteoporotic (n-34)		Non-osteoporosis (*n* = 73)		*P*-value
	Number	Percent	Number	Percent	
*Sex*					0.032
Male	7	20.59	31	42.47	
Female	27	79.41	42	57.53	
*Educational status*					0.001
Illiterate	23	67.65	13	17.81	
Non-academic	10	29.41	49	67.12	
Academic	1	2.94	11	15.07	
*Marital status*					0.023
Married	26	76.47	68	93.15	
Single	8	23.53	5	6.85	
*Employment status*					0.004
Employee	0	0.00	7	9.59	
Free job	1	2.94	18	21.66	
Unemployed	4	11.76	3	4.11	
Housewife	25	73.53	38	52.05	
Retired	4	11.76	7	9.59	
*Menopausal status*					0.018
Yes	25	92.59	28	66.67	
No	2	7.41	14	33.33	
*Taking corticosteroids*					0.028
Yes	6	17.65	3	4.11	
No	28	82.35	70	95.89	
*Smoking*					0.552
Yes	6	17.65	9	12.33	
No	28	82.35	64	87.67	
*Alcohol consumption*					0.305
Yes	0	0.00	4	5.48	
No	34	100	69	94.52	

Continuous variables	Mean	SD	Mean	SD	*P*-value
Age (y)	67.91	7.02	59.19	5.12	0.001
Body mass index	27.46	3.26	26.08	2.39	0.015

The results of Table 2 about comparison of the posoperational clinical and paraclinical charatecristics of the study population reveal that there was a significant difference between two groups in the loosening of instrument (*p* = 0.039).

**Table 2:** Comparison of the posoperational clinical and paraclinical charatecristics of the study population using the Fisher exact test

Variables	Osteoporotic (n-34)		Non-osteoporosis (*n* = 73)		*P*-value
	Number	Percent	Number	Percent	
*Infection*					1.000
Yes	2	5.88	4	5.48	
No	32	94.12	69	94.52	
*Loosening of instrument*					0.039
Yes	9	26.47	7	9.59	
No	25	73.53	66	90.41	
*Displacement of instrument*					0.318
Yes	1	2.94	0	0.00	
No	33	97.06	73	100	
*Fracture of instrument*					0.305
Yes	0	0.00	4	5.48	
No	34	100	69	94.52	
*Fusion*					0.305
1	1	2.94	6	8.22	
2	2	5.88	8	10.96	
3	3	8.82	15	20.55	
4	7	20.59	11	15.07	
5	21	61.76	33	45.21	

Based on Table 3, the results of the Mann–Whitney test indicate that there was a significant difference between two groups in the postoperative pain intensity (*p* = 0.007), fusion (*p* = 0.047), and neurogenic claudication (*p* = 0.003).

**Table 3:** Comparison of the clinical and paraclinical charatecristics of the study population

Variables	Osteoporotic (n-34)		Non-osteoporosis (*n* = 73)		*P*-value†	*P*-value‡
	Mean	SD	Mean	SD		
Preoperative pain intensity	5.44	2.16	5.04	1.54	0.127	0.215
Postoperative pain intensity	1.82	1.26	1.17	1.03	0.007	0.991
Fusion	3.23	1.01	2.77	1.02	0.047	0.137
Neurogenic claudication	57.05	38.41	90.41	62.16	0.003	0.201

^†^Mann–Whitney test (unadjusted analysis)

^‡^Multiple ANCOVA test adjusted for potential confounders including sex, educational level, marital status, occupation, monopausal status, taking corticosteroid, age, and BMI

Also, the findings of comparison of the pre and postoperative clinical and paraclinical charatecristics of the study population using the Rank Wilcoxson test show that in terms of preoperative and postoperative pain intensity, a significant difference was observed among the osteoporotic group patients (*p* = 0.001) and the non-osteoporotic group patients (*p* = 0.001) (Table 4).

**Table 4:** Comparison of the pre and postoperational clinical and paraclinical charatecristics of the study population using the Rank Wilcoxson test

Variables	Preoperative		Postoperative		*P*-value
	Mean	SD	Mean	SD	
Pain intensity in osteoporotic group	5.44	2.16	1.82	1.26	0.001
Pain intensity in non-osteoporotic group	5.04	1.54	1.17	1.03	0.001

## Discussion

The aim of the present study was designed to evaluate the clinical outcome of lumbar spinal canal stenosis after instrumentation in patients with and without osteoporosis.

The results of our study indicated that there was a significant difference in the mean age between two groups. The group with osteoporosis had a higher mean age. Aging has been confirmed as a risk factor for osteoporosis in previous studies [14, 15]. This finding was consistent with the results of a study in South Korea; in that study, the prevalence of osteoporosis was significantly increased with age. Also, patients > 50 years old (especially women) who need spinal surgery often suffer from osteoporosis, and the number of spinal surgeries in these patients increases [4]. Not only do bones lose density with age, but bone formation also decreases significantly. These changes predispose the elderly, especially women, to osteoporosis [14]. Therefore, according to these findings, it is recommended to treat osteoporosis for these patients after surgery, especially in women over 50 years [4].

In this study, a significant difference was observed in the menopausal status between two groups; in the group with osteoporosis, most of the studied women were menopause. Osteoporosis has been identified as the most common disease in postmenopausal women [16]; approximately 10 percent of the world's population and 30 percent of postmenopausal women suffer from osteoporosis [17, 18]. Risk factors for osteoporosis are divided into two categories: modifiable factors (weight, smoking, alcohol consumption, sedentary lifestyle, Inadequate nutritional absorption, stress, and air pollution) and non-modifiable factors (older age, sex, race, history of fall, prior fracture, and family history of osteoporosis) [19]. The study of Bijelic et al. also mentioned genetic risk factors and environmental risk factors (smoking, alcohol, and coffee consumption) that alone or together can reduce osteoporosis and lead to osteoporosis in postmenopausal women [20]. So, considering that in our study in the group with osteoporosis, most of the studied women were menopausal, it is important to pay attention to the modifiable factors of osteoporosis in this group of population. Also, it sounds that planning and implementing gender-based interventions concerning modifiable factors can be useful for them.

In the current study, there was a significant difference in the history of taking corticosteroids in the two groups; the group with osteoporosis took more corticosteroids. This finding was consistent with other studies [21, 22]. Osteoporosis is one of the most serious side effects of long-term use of corticosteroids. After initiating corticosteroid therapy, an increase in bone resorption due to suppression of the osteoblastic process occurs [22]. Osteoporosis caused by corticosteroids is the most common form of secondary osteoporosis. Bone loss and increased fracture rate occur early after the start of corticosteroid treatment and after that, complications continue depending on the dose and duration of treatment [21]. Therefore, it is essential to take corticosteroids only in necessary cases with the minimum dose and minimum treatment period. In patients who use corticosteroids for a long time, bone densitometry should be performed and this test should be repeated at regular intervals.

In the present study, a significant difference was observed in the mean BMI between two groups; the group with osteoporosis had a higher mean BMI. The results of the previous studies reveal that although the most important indicator of bone tissue metabolic process, regardless of menopause, is BMI [23–25], contradictory evidence is available about the relationship between BMI and osteoporosis; For example, some studies have shown a negative relationship between weight and body fat mass with bone density [26–28], while some other studies have indicated a positive relationship between these two variables [29, 30]. Therefore, further studies are needed in this field. The existing evidence suggests that the relationship between BMI and bone density in women is more U-shaped than linear [31, 32]. It seems that given that weight is one of the modifiable risk factors in reducing osteoporosis, it is necessary to advise patients to maintain their normal weight [33].

In the present study, there was a significant difference between screw loosening in two groups; so that the group with osteoporosis became looser. In our study, in the group with osteoporosis, 9 patients (26.47%) had loosened and 1 patient (2.94%) had displacement, but no screw fractures were reported. In the group without osteoporosis, 7 patients (9.59%) had loosening and 4 patients (5.48%) had fractures, but no screw displacement was reported. The results of a study by Wu et al. (2012) in China, which aimed to compare the degree of loosening of the screw and the clinical outcome of expandable pedicular screws (EPS) with conventional pedicular screws (CPS) in patients with spinal stenosis who had osteoporosis, showed that in the EPS group, 20 screws (4.1%) were loosened in 6 patients and 2 screws (0.4%) were fractured. In the CPS group, 48 screws (12.9%) were loosened in 15 patients but no screw fractures were reported. In the EPS group, two screws were fractured without causing any neurological complications. The results of the study illustrated that EPS can reduce the risk of screw loosening and improve fixation in patients with osteoporosis [34]**.**

In our study, there was no significant difference in the presence of infection between two groups; in the group with osteoporosis, two patients (5.88%) and the group without osteoporosis, four patients (5.48%) became infected. The incidence of postoperative infection in both groups was similar to the study of Schimmel et al. [16]. It should be noted that all cases of postoperative infection in this study were superficial infections and were treated as outpatients. In the present study, the overall fusion rate was 38.23% in the group with osteoporosis and 54.79% in the group without osteoporosis. In the study of Bjerke et al., the complications related to osteoporosis were higher in patients with lower bone density, which included a lower fusion rate [17].

In our study, there was a significant difference in the mean postoperative pain intensity between two groups; the mean postoperative pain was higher in the group with osteoporosis. It seems that the presence of osteoporosis and its effects on bone density in these patients reduces the resistance of the vertebrae to the weight of the body that is transmitted to the spine, which could be a possible reason for the higher severity of pain in this group of patients. Also, the there was a significant difference between two groups in the preoperative and postoperative pain intensity. In such a way that the mean of postoperative pain intensity was significantly reduced in both groups. It seems that the reduction in pain in both groups can be due to the removal of spinal canal stenosis and fusion in the spine.

In the study of Wu, the fusion rate in the group of patients with conventional pedicular screws (CPS) was 80.5%, which is much higher than in the present study. In the group with osteoporosis who had a fusion, given the fusion was graded, the highest frequency is related to grade 4 according to Glassman criterion, 20.59%, which is the weakest degree of fusion. In the present study, there was no statistically significant difference between the two groups in terms of fusion degree, which may be due to drug treatment of osteoporosis in patients after surgery and follow-up of their treatment. Considering the lower rate of fusion in our study than in other studies, it seems that need to review the process of fusion in patients at all stages, including preparation of the fusion bed, type of eclipse, volume, and fusion sites.

In our study, a significant difference was observed in the mean neurogenic claudication between the groups; the without osteoporosis group had a higher mean neurogenic claudication. According to the study of Lee et al., the higher prevalence may be because difficulty in walking and physical activity because of claudication is associated with a decrease in bone density [18].

The results of this study should be interpreted in light of some limitations. One limitation is related to the patient's unwillingness to perform CT scan to more accurately assess the fusion rate. Another limitation of our study was that we prescribed the most cost-effective treatment regimen for osteoporosis. The reasons for this were that the lack of insurance coverage and the high cost of other treatment regimens of osteoporosis. It sounds that by prescribing different regimens for the treatment of osteoporosis, it will be possible to compare their therapeutic effects and choose the best regimen to increase the rate of fusion.

## Conclusion

This study provides evidence that there is no significant difference in the clinical outcomes of lumbar spine instrumentation due to spinal canal stenosis in patients with and without osteoporosis. Because of the high cost of specific instrumentation developed for patients with osteoporosis and their unavailability for all patients, it seems that the use of conventional instrumentation along with complete treatment of osteoporosis can help improve the clinical outcome of surgery in these patients.

## Data Availability

The analyzed dataset in this study is available from the first author on reasonable request.
